# 9-Hexyl-3-iodo-9*H*-carbazole

**DOI:** 10.1107/S1600536809054415

**Published:** 2009-12-24

**Authors:** Wen-Qian Geng, Guo-Yi Xu, Hong-Ping Zhou

**Affiliations:** a Department of Chemistry, Anhui University, Hefei 230039, People’s Republic of China, and Key Laboratory of Functional Inorganic Materials Chemistry, Hefei 230039, People’s Republic of China

## Abstract

In the title mol­ecule, C_18_H_20_IN, the tricyclic carbazole system is essentially planar with the two outer rings forming a dihedral angle of 0.43 (8)°. The crystal packing exhibits no short inter­molecular contacts.

## Related literature

For the crystal structures of related carbazole derivatives, see: Zhou *et al.* (2007 ▶, 2008 ▶); Chen *et al.* (2009 ▶).
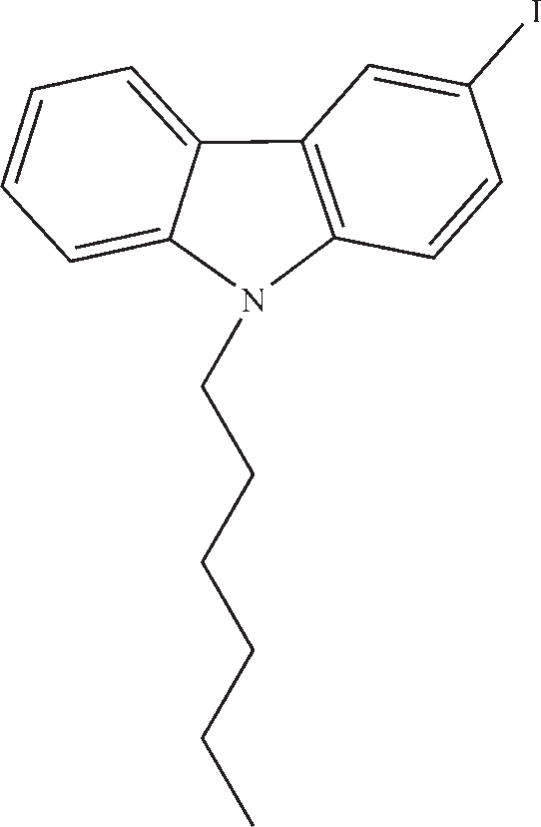


## Experimental

### 

#### Crystal data

C_18_H_20_IN
*M* *_r_* = 377.25
Monoclinic, 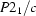

*a* = 10.7105 (2) Å
*b* = 4.6816 (10) Å
*c* = 33.9661 (18) Å
β = 105.106 (8)°
*V* = 1644.3 (4) Å^3^
*Z* = 4
Mo *K*α radiation
μ = 1.94 mm^−1^
*T* = 298 K
0.30 × 0.20 × 0.20 mm

#### Data collection

Bruker SMART CCD area-detector diffractometer
Absorption correction: multi-scan (*SADABS*; Sheldrick, 1996 ▶) *T*_min_ = 0.594, *T*_max_ = 0.698
13335 measured reflections
2904 independent reflections
2123 reflections with *I* > 2σ(*I*)
*R*_int_ = 0.024

#### Refinement

*R*[*F* ^2^ > 2σ(*F* ^2^)] = 0.040
*wR*(*F* ^2^) = 0.164
*S* = 1.14
2904 reflections
182 parameters
13 restraints
H-atom parameters constrained
Δρ_max_ = 1.03 e Å^−3^
Δρ_min_ = −0.61 e Å^−3^

### 

Data collection: *SMART* (Bruker, 2002 ▶); cell refinement: *SAINT* (Bruker, 2002 ▶); data reduction: *SAINT*; program(s) used to solve structure: *SHELXS97* (Sheldrick, 2008 ▶); program(s) used to refine structure: *SHELXL97* (Sheldrick, 2008 ▶); molecular graphics: *SHELXTL* (Sheldrick, 2008 ▶); software used to prepare material for publication: *SHELXTL*.

## Supplementary Material

Crystal structure: contains datablocks I, global. DOI: 10.1107/S1600536809054415/cv2679sup1.cif

Structure factors: contains datablocks I. DOI: 10.1107/S1600536809054415/cv2679Isup2.hkl

Additional supplementary materials:  crystallographic information; 3D view; checkCIF report

## Figures and Tables

**Fig. 1.**
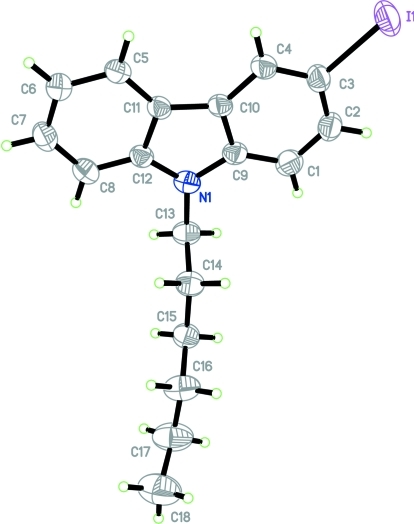
Molecular structure of (I) with displacement ellipsoids shown at 30% probability level.

## supplementary crystallographic information

### Comment

In continuation of our study of carbazole derivatives (Zhou *et al.*, 2007, 2008) we present here the title compound, (I). In (I) (Fig. 1), the bond lengths and angles show normal values comparable with those observed in the related compounds (Zhou *et al.*, 2008; Chen *et al.*, 2009). Two outer rings form a dihedral angle of 0.43 (8) °. The crystal packing exhibits no essentially short intermolecular contacts.

### Experimental

A 50 ml round bottom flask was charged with 9-hexlylcarbazole (2.51 g, 10 mmol) and 10 ml e thanol. ICl (2.50 g, 15 mmol, dissolved in 4 ml ethanol) was added to the stirring solution at 343 k after 9-(hex-1-yl)-3-iodocarbazole was completely dissolved. At the end of the reaction was judged by TLC analysis after 2 h. The solution was filtered through filter paper and precipitates were obtained as a light blue solid in 89% (4.48 g) yield.

### Refinement

All hydrogen atoms were placed in geometrically idealized positions and constrained to ride on their parent atoms, with C—H = 0.93 - 0.97 Å and *U*_iso_(H) = 1.2-1.5*U*_eq_(C).

### Crystal data

**Table:**

C_18_H_20_IN	*F*(000) = 752
*M**_r_* = 377.25	*D*_x_ = 1.524 Mg m^−^^3^
Monoclinic, *P*2_1_/*c*	Mo *K*α radiation, λ = 0.71069 Å
Hall symbol: -P 2ybc	Cell parameters from 4151 reflections
*a* = 10.7105 (2) Å	θ = 2.6–23.7°
*b* = 4.6816 (10) Å	µ = 1.94 mm^−^^1^
*c* = 33.9661 (18) Å	*T* = 298 K
β = 105.106 (8)°	Bar, colourless
*V* = 1644.3 (4) Å^3^	0.30 × 0.20 × 0.20 mm
*Z* = 4	

### Data collection

**Table:**

Bruker SMART CCD area-detector diffractometer	2904 independent reflections
Radiation source: fine-focus sealed tube	2123 reflections with *I* > 2σ(*I*)
graphite	*R*_int_ = 0.024
phi and ω scans	θ_max_ = 25.0°, θ_min_ = 2.0°
Absorption correction: multi-scan (*SADABS*; Sheldrick, 1996)	*h* = −12→12
*T*_min_ = 0.594, *T*_max_ = 0.698	*k* = −5→5
13335 measured reflections	*l* = −40→40

### Refinement

**Table:**

Refinement on *F*^2^	Primary atom site location: structure-invariant direct methods
Least-squares matrix: full	Secondary atom site location: difference Fourier map
*R*[*F*^2^ > 2σ(*F*^2^)] = 0.040	Hydrogen site location: inferred from neighbouring sites
*wR*(*F*^2^) = 0.164	H-atom parameters constrained
*S* = 1.14	*w* = 1/[σ^2^(*F*_o_^2^) + (0.1*P*)^2^] where *P* = (*F*_o_^2^ + 2*F*_c_^2^)/3
2904 reflections	(Δ/σ)_max_ = 0.002
182 parameters	Δρ_max_ = 1.03 e Å^−^^3^
13 restraints	Δρ_min_ = −0.61 e Å^−^^3^

### Special details

**Table:**

Geometry. All e.s.d.'s (except the e.s.d. in the dihedral angle between two l.s. planes) are estimated using the full covariance matrix. The cell e.s.d.'s are taken into account individually in the estimation of e.s.d.'s in distances, angles and torsion angles; correlations between e.s.d.'s in cell parameters are only used when they are defined by crystal symmetry. An approximate (isotropic) treatment of cell e.s.d.'s is used for estimating e.s.d.'s involving l.s. planes.
Refinement. Refinement of *F*^2^ against ALL reflections. The weighted *R*-factor *wR* and goodness of fit *S* are based on *F*^2^, conventional *R*-factors *R* are based on *F*, with *F* set to zero for negative *F*^2^. The threshold expression of *F*^2^ > σ(*F*^2^) is used only for calculating *R*-factors(gt) *etc*. and is not relevant to the choice of reflections for refinement. *R*-factors based on *F*^2^ are statistically about twice as large as those based on *F*, and *R*- factors based on ALL data will be even larger.

### Fractional atomic coordinates and isotropic or equivalent isotropic displacement parameters (Å^2^)

**Table:**

	*x*	*y*	*z*	*U*_iso_*/*U*_eq_	
I1	−0.28721 (3)	0.22226 (10)	0.854577 (12)	0.1016 (3)	
N1	0.2490 (4)	0.7102 (7)	0.85154 (11)	0.0642 (10)	
C1	0.0702 (5)	0.6986 (10)	0.88720 (15)	0.0764 (14)	
H1	0.1078	0.8288	0.9075	0.092*	
C2	−0.0462 (4)	0.5812 (12)	0.88584 (12)	0.0784 (13)	
H2	−0.0887	0.6318	0.9054	0.094*	
C3	−0.1033 (4)	0.3863 (11)	0.85567 (12)	0.0691 (11)	
C4	−0.0449 (4)	0.3024 (9)	0.82657 (13)	0.0642 (11)	
H4	−0.0839	0.1699	0.8068	0.077*	
C5	0.1625 (4)	0.2277 (9)	0.76745 (13)	0.0642 (12)	
H5	0.0931	0.1082	0.7561	0.077*	
C6	0.2669 (5)	0.2482 (9)	0.75017 (15)	0.0733 (14)	
H6	0.2675	0.1406	0.7272	0.088*	
C7	0.3691 (4)	0.4269 (11)	0.76693 (14)	0.0752 (12)	
H7	0.4373	0.4394	0.7548	0.090*	
C8	0.3731 (4)	0.5878 (10)	0.80120 (12)	0.0677 (11)	
H8	0.4433	0.7053	0.8125	0.081*	
C9	0.1323 (4)	0.6213 (9)	0.85775 (11)	0.0588 (10)	
C10	0.0761 (3)	0.4209 (9)	0.82713 (10)	0.0550 (9)	
C11	0.1639 (3)	0.3892 (9)	0.80204 (10)	0.0543 (9)	
C12	0.2694 (3)	0.5687 (8)	0.81814 (10)	0.0544 (9)	
C13	0.3408 (4)	0.9010 (10)	0.87894 (12)	0.0705 (11)	
H13A	0.2938	1.0335	0.8917	0.085*	
H13B	0.3879	1.0110	0.8633	0.085*	
C14	0.4357 (5)	0.7305 (8)	0.91164 (16)	0.0714 (13)	
H14A	0.3878	0.6282	0.9278	0.086*	
H14B	0.4777	0.5899	0.8985	0.086*	
C15	0.5381 (4)	0.9110 (11)	0.93974 (13)	0.0745 (12)	
H15A	0.5777	1.0328	0.9233	0.089*	
H15B	0.4967	1.0340	0.9555	0.089*	
C16	0.6420 (6)	0.7450 (10)	0.96843 (19)	0.096 (2)	
H16A	0.6773	0.6077	0.9528	0.115*	
H16B	0.6037	0.6386	0.9868	0.115*	
C17	0.7505 (5)	0.9222 (13)	0.99323 (18)	0.118 (2)	
H17A	0.7797	1.0512	0.9751	0.142*	
H17B	0.7178	1.0378	1.0121	0.142*	
C18	0.8625 (7)	0.7562 (13)	1.0167 (3)	0.144 (3)	
H18A	0.9236	0.8826	1.0340	0.216*	
H18B	0.9030	0.6609	0.9983	0.216*	
H18C	0.8338	0.6170	1.0332	0.216*	

### Atomic displacement parameters (Å^2^)

**Table:**

	*U*^11^	*U*^22^	*U*^33^	*U*^12^	*U*^13^	*U*^23^
I1	0.0687 (3)	0.1321 (5)	0.1158 (4)	−0.00227 (17)	0.0452 (3)	0.0162 (2)
N1	0.054 (2)	0.068 (2)	0.065 (2)	−0.0032 (16)	0.0064 (16)	−0.0033 (15)
C1	0.075 (3)	0.086 (3)	0.066 (3)	0.011 (2)	0.015 (2)	−0.003 (2)
C2	0.073 (3)	0.104 (4)	0.063 (2)	0.018 (3)	0.027 (2)	0.009 (3)
C3	0.055 (2)	0.088 (3)	0.068 (2)	0.012 (2)	0.0223 (19)	0.017 (2)
C4	0.055 (2)	0.074 (3)	0.061 (2)	0.002 (2)	0.0105 (18)	0.0064 (19)
C5	0.052 (2)	0.073 (3)	0.064 (2)	−0.0023 (18)	0.0103 (19)	−0.0076 (18)
C6	0.066 (3)	0.086 (4)	0.069 (3)	0.001 (2)	0.021 (2)	−0.009 (2)
C7	0.058 (2)	0.087 (3)	0.086 (3)	0.003 (2)	0.029 (2)	0.008 (3)
C8	0.054 (2)	0.069 (3)	0.079 (3)	−0.006 (2)	0.0164 (19)	0.002 (2)
C9	0.055 (2)	0.064 (2)	0.054 (2)	0.009 (2)	0.0071 (17)	0.0056 (19)
C10	0.046 (2)	0.062 (2)	0.0530 (19)	0.0080 (19)	0.0067 (15)	0.0101 (18)
C11	0.0456 (19)	0.060 (2)	0.055 (2)	0.0056 (18)	0.0092 (15)	0.0071 (17)
C12	0.0479 (19)	0.057 (2)	0.0555 (19)	0.0039 (18)	0.0089 (15)	0.0050 (17)
C13	0.071 (3)	0.062 (3)	0.071 (2)	−0.005 (2)	0.006 (2)	−0.006 (2)
C14	0.071 (3)	0.062 (3)	0.077 (3)	−0.004 (2)	0.010 (2)	−0.0026 (19)
C15	0.068 (3)	0.079 (3)	0.075 (3)	−0.015 (2)	0.014 (2)	−0.009 (2)
C16	0.099 (5)	0.080 (4)	0.089 (4)	0.006 (3)	−0.009 (3)	−0.008 (2)
C17	0.118 (4)	0.090 (4)	0.121 (4)	0.005 (4)	−0.014 (3)	−0.021 (3)
C18	0.116 (6)	0.135 (6)	0.155 (6)	0.007 (4)	−0.011 (5)	−0.028 (4)

### Geometric parameters (Å, °)

**Table:**

I1—C3	2.106 (4)	C9—C10	1.413 (6)
N1—C12	1.379 (5)	C10—C11	1.431 (5)
N1—C9	1.385 (6)	C11—C12	1.400 (5)
N1—C13	1.467 (5)	C13—C14	1.522 (6)
C1—C2	1.351 (7)	C13—H13A	0.9700
C1—C9	1.386 (7)	C13—H13B	0.9700
C1—H1	0.9300	C14—C15	1.511 (6)
C2—C3	1.389 (7)	C14—H14A	0.9700
C2—H2	0.9300	C14—H14B	0.9700
C3—C4	1.358 (6)	C15—C16	1.492 (7)
C4—C10	1.405 (6)	C15—H15A	0.9700
C4—H4	0.9300	C15—H15B	0.9700
C5—C11	1.394 (6)	C16—C17	1.496 (8)
C5—C6	1.395 (7)	C16—H16A	0.9700
C5—H5	0.9300	C16—H16B	0.9700
C6—C7	1.378 (7)	C17—C18	1.477 (7)
C6—H6	0.9300	C17—H17A	0.9700
C7—C8	1.378 (6)	C17—H17B	0.9700
C7—H7	0.9300	C18—H18A	0.9600
C8—C12	1.381 (5)	C18—H18B	0.9600
C8—H8	0.9300	C18—H18C	0.9600
			
C12—N1—C9	108.9 (3)	C8—C12—C11	122.1 (4)
C12—N1—C13	126.2 (4)	N1—C13—C14	110.7 (4)
C9—N1—C13	124.6 (4)	N1—C13—H13A	109.5
C2—C1—C9	118.8 (5)	C14—C13—H13A	109.5
C2—C1—H1	120.6	N1—C13—H13B	109.5
C9—C1—H1	120.6	C14—C13—H13B	109.5
C1—C2—C3	121.1 (4)	H13A—C13—H13B	108.1
C1—C2—H2	119.4	C15—C14—C13	113.9 (4)
C3—C2—H2	119.4	C15—C14—H14A	108.8
C4—C3—C2	121.9 (4)	C13—C14—H14A	108.8
C4—C3—I1	119.2 (4)	C15—C14—H14B	108.8
C2—C3—I1	118.9 (3)	C13—C14—H14B	108.8
C3—C4—C10	118.3 (4)	H14A—C14—H14B	107.7
C3—C4—H4	120.9	C16—C15—C14	114.6 (4)
C10—C4—H4	120.9	C16—C15—H15A	108.6
C11—C5—C6	118.9 (4)	C14—C15—H15A	108.6
C11—C5—H5	120.6	C16—C15—H15B	108.6
C6—C5—H5	120.6	C14—C15—H15B	108.6
C7—C6—C5	120.4 (4)	H15A—C15—H15B	107.6
C7—C6—H6	119.8	C15—C16—C17	114.6 (4)
C5—C6—H6	119.8	C15—C16—H16A	108.6
C6—C7—C8	121.8 (4)	C17—C16—H16A	108.6
C6—C7—H7	119.1	C15—C16—H16B	108.6
C8—C7—H7	119.1	C17—C16—H16B	108.6
C7—C8—C12	117.7 (4)	H16A—C16—H16B	107.6
C7—C8—H8	121.1	C18—C17—C16	114.5 (5)
C12—C8—H8	121.1	C18—C17—H17A	108.6
N1—C9—C1	130.8 (4)	C16—C17—H17A	108.6
N1—C9—C10	108.4 (4)	C18—C17—H17B	108.6
C1—C9—C10	120.8 (4)	C16—C17—H17B	108.6
C4—C10—C9	119.1 (4)	H17A—C17—H17B	107.6
C4—C10—C11	134.2 (4)	C17—C18—H18A	109.5
C9—C10—C11	106.7 (3)	C17—C18—H18B	109.5
C5—C11—C12	119.0 (4)	H18A—C18—H18B	109.5
C5—C11—C10	133.9 (4)	C17—C18—H18C	109.5
C12—C11—C10	107.1 (3)	H18A—C18—H18C	109.5
N1—C12—C8	128.9 (4)	H18B—C18—H18C	109.5
N1—C12—C11	109.0 (3)		
			
C9—C1—C2—C3	0.1 (7)	C6—C5—C11—C10	180.0 (4)
C1—C2—C3—C4	0.7 (7)	C4—C10—C11—C5	−0.1 (7)
C1—C2—C3—I1	−178.2 (3)	C9—C10—C11—C5	−179.3 (4)
C2—C3—C4—C10	−0.8 (6)	C4—C10—C11—C12	179.4 (4)
I1—C3—C4—C10	178.1 (3)	C9—C10—C11—C12	0.2 (4)
C11—C5—C6—C7	−0.5 (7)	C9—N1—C12—C8	−179.2 (4)
C5—C6—C7—C8	0.8 (7)	C13—N1—C12—C8	−5.2 (6)
C6—C7—C8—C12	−1.1 (7)	C9—N1—C12—C11	0.9 (4)
C12—N1—C9—C1	179.5 (4)	C13—N1—C12—C11	175.0 (4)
C13—N1—C9—C1	5.4 (7)	C7—C8—C12—N1	−178.6 (4)
C12—N1—C9—C10	−0.8 (4)	C7—C8—C12—C11	1.2 (6)
C13—N1—C9—C10	−175.0 (3)	C5—C11—C12—N1	178.9 (3)
C2—C1—C9—N1	178.8 (4)	C10—C11—C12—N1	−0.7 (4)
C2—C1—C9—C10	−0.8 (6)	C5—C11—C12—C8	−1.0 (6)
C3—C4—C10—C9	0.1 (5)	C10—C11—C12—C8	179.5 (4)
C3—C4—C10—C11	−179.1 (4)	C12—N1—C13—C14	−84.0 (5)
N1—C9—C10—C4	−179.0 (3)	C9—N1—C13—C14	89.2 (5)
C1—C9—C10—C4	0.7 (6)	N1—C13—C14—C15	176.8 (4)
N1—C9—C10—C11	0.4 (4)	C13—C14—C15—C16	−172.3 (5)
C1—C9—C10—C11	−179.9 (4)	C14—C15—C16—C17	174.0 (6)
C6—C5—C11—C12	0.6 (6)	C15—C16—C17—C18	−170.8 (7)
